# Nanofiber Systems as Herbal Bioactive Compounds Carriers: Current Applications in Healthcare

**DOI:** 10.3390/pharmaceutics14010191

**Published:** 2022-01-14

**Authors:** Kathya Huesca-Urióstegui, Elsy J. García-Valderrama, Janet A. Gutierrez-Uribe, Marilena Antunes-Ricardo, Daniel Guajardo-Flores

**Affiliations:** Tecnológico de Monterrey, Centro de Biotecnologia-FEMSA., Av. Eugenio Garza Sada 2501 Sur, Monterrey 64849, NL, Mexico; khuescau95@gmail.com (K.H.-U.); a01270260@itesm.mx (E.J.G.-V.); jagu@tec.mx (J.A.G.-U.)

**Keywords:** nanofibers, bioactive compounds, herbal extracts, healthcare, in vivo studies

## Abstract

Nanofibers have emerged as a potential novel platform due to their physicochemical properties for healthcare applications. Nanofibers’ advantages rely on their high specific surface-area-to-volume and highly porous mesh. Their peculiar assembly allows cell accommodation, nutrient infiltration, gas exchange, waste excretion, high drug release rate, and stable structure. This review provided comprehensive information on the design and development of natural-based polymer nanofibers with the incorporation of herbal medicines for the treatment of common diseases and their in vivo studies. Natural and synthetic polymers have been widely used for the fabrication of nanofibers capable of mimicking extracellular matrix structure. Among them, natural polymers are preferred because of their biocompatibility, biodegradability, and similarity with extracellular matrix proteins. Herbal bioactive compounds from natural extracts have raised special interest due to their prominent beneficial properties in healthcare. Nanofiber properties allow these systems to serve as bioactive compound carriers to generate functional matrices with antimicrobial, anti-inflammatory, antioxidant, antiseptic, anti-viral, and other properties which have been studied in vitro and in vivo, mostly to prove their wound healing capacity and anti-inflammation properties.

## 1. Introduction

Nanotechnology has enabled the development of new technology to generate functional nanomaterials capable of carrying drugs in the form of dendrimers, liposomes, nanoshells, emulsions, nanotubes, and nanofibers for the treatment of certain diseases. Among them, nanofibers have offered a great potential application as a drug carrier system of synthetic or natural compounds that have poor bioavailability [1]. They are polymeric fibers with diameters in the range of nanometers and lengths in meters. Due to their structure and characteristics such as small pore size, surface-area-to-volume ratio, high tensile strength, and low coefficient of thermal expansion, nanofibers mimic the natural extracellular matrix (ECM), withstanding biological processes, enhancing cell attachment, migration, and facilitating nutrient transport [2,3].

The use of isolated bioactive compounds, obtained from medicinal plants, known as synthetic medicinal plants, represents high cost and has been shown to induce negative side effects in humans. These negative effects have been attributed to the purification process that can produce toxic byproducts [4,5]. In the last years, the interest in the use of medicinal plants, herbal extracts, and natural bioactive compounds, instead of synthetic medicinal plants, has grown between scientists as a strategy to develop treatments safer and accessible for different diseases, to rescue ancient knowledge about herbal medicine. Herbal extracts are preparations obtained from natural sources, mainly plants, which contain bioactive ingredients or components that exert effective actions on different biological molecular targets related to pathophysiological alterations of different diseases. The most studied biological activities of herbal extracts are antimicrobial, anti-virus, anti-inflammatory, and anticancer agents [6,7,8].

This review will provide and discuss the current strategies for the design and development of natural-based polymer nanofibers with the incorporation of herbal extracts for the treatment of common diseases and their in vivo studies. Specifically, this review will show the therapeutic effect of natural bioactive compounds in active nanofibers summarizing their usage in the healthcare field as functional matrices in wound dressings, tissue engineering, and drug delivery systems. Finally, some in vivo studies were added in order to compare the wound healing potential of these nanofibers to the commercially available products. The novelty of this study is that it provides a panoramic and integrated view of the use of technology, especially nanotechnology, and ancestral medicine for the development of delivery systems with health applications that simulate different benefits through a combination unique among various active components present in each of the extracts.

## 2. Methods for Nanofiber Fabrication

Several techniques for the production of nanofibers have been developed in order to reduce production time, to include more and better materials, and to lower costs, among other characteristics [9,10,11,12]. The most used techniques are electrospinning (e-spinning), centrifugal spinning (Forcespinning^TM^), solution blow spinning, and carbon dioxide (CO_2_) laser supersonic drawing. In this section, their main characteristics and advantages will be discussed.

### 2.1. Electrospinning (E-Spinning)

**Figure 1:**
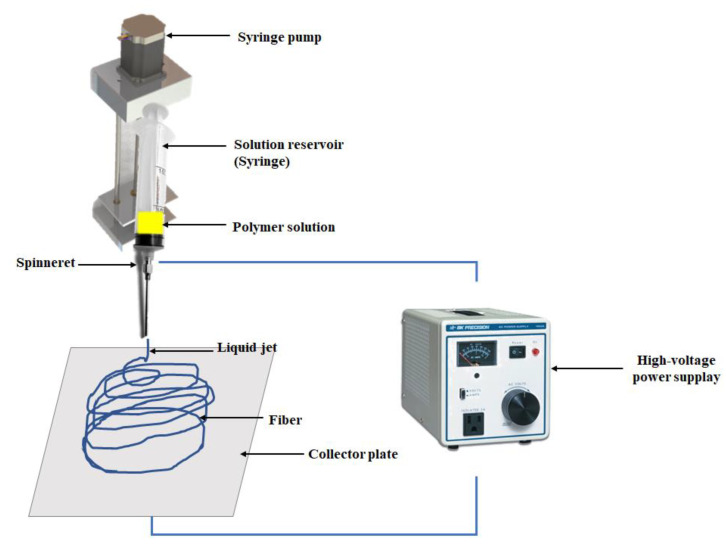
Schematic representation of electrospinning.

Among the current strategies to produce nanofibers, electrospinning (e-spinning) has been the most widely used technique over the last years. Electrospinning has offered several advantages such as easy control for fiber diameter, morphology, surface characteristics, mesh porosity, and the possibility of getting fibers with diameters ranging from tens of nanometers to several micrometers [13,14]. Electrospinning involves an electrohydrodynamic process, where a high-voltage electric field is generated between the injection needle and the collecting screen, then a liquid droplet is electrified to generate a jet, followed by stretching and elongation to generate fiber(s). The major components required for the e-spinning technique include a high-voltage power supply, a syringe pump, a spinneret, and a conductive collector (Figure 1) [14,15].

The physical characteristics of the nanofibers depends mainly on three factors: (i) the polymer solution parameters (viscosity, surface tension, conductivity, polymer molecular weight, solvent volatility, dielectric constant), (ii) technical parameters (voltage, volume flow rate conductivity, tip-to-collector distance, collector geometry, needle diameter), and (iii) environmental conditions (temperature and humidity) [16]. Thus, it allows the fabrication of functional and cost-effective fiber membranes that, alone or in combination of biopolymers with other components, show good structural properties with various compositions, morphologies, and bioactivities [17,18].

With the development of new materials and the evolution of the technology, it will be possible to develop three-dimensional systems as complex and versatile as required using the e-spinning technique. It will be possible to generate scaffolds as substitutes for human tissues, systems for the controlled and sustained delivery of drugs, medical devices, and novel cosmetic products that could even replace some surgeries. Despite its wide applications, the electrospinning technique presents diverse limitations such as low yield, use of specialized equipment, high electrical potential, electrically conductive targets, and high costs [19]. However, a major limitation of electrospinning is the difficulty to produce fibers with diameters down to the nanometer scale and the degradation of some polymers or bioactive compounds as a result of the extended heating [13,15]. To overcome these limitations, a wide number of new technologies have been developed to improve the total field.

### 2.2. Centrifugal Spinning (Forcespinning^TM^)

**Figure 2:**
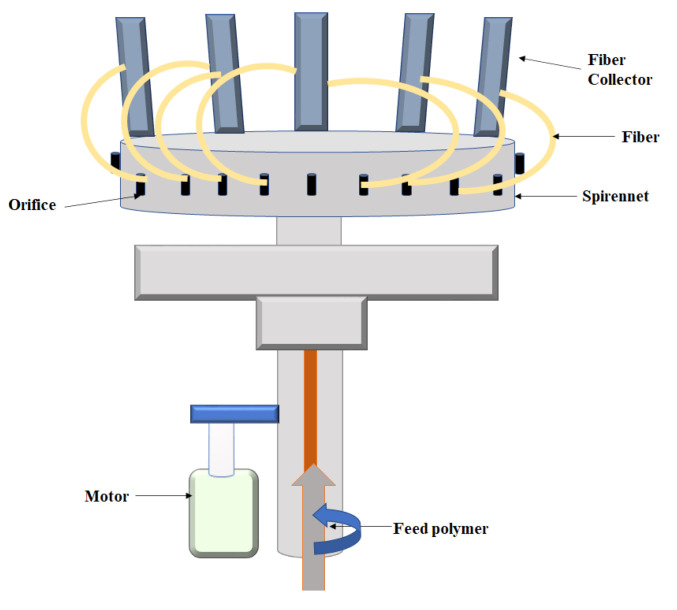
Schematic representation of centrifugal spinning.

Centrifugal spinning or Forcespinning^TM^ has emerged as a novel strategy for the fabrication of nanofibers; this technique is considered a non-electrical nanofiber spinning because it uses centrifugal forces to form nanofibers (average diameter ranging between 130–300 nm) instead of high voltage conditions [20,21]. Unlike e-spinning, the method consists of ejecting a polymer solution through thin needles which rotate at high-speed conditions; thus, the centrifugal force exceeds the surface tension of the spinning solutions and produces solidification, stretching of the polymer, and the evaporation of the solvent, allowing the collection of the nanofibers on the walls of a cylindrical collector (Figure 2) [22,23].

In addition to the centrifugal force, the nanofibers are also influenced by the air friction force, rheological force, surface tension, and gravitational force [24]. In Table 1, the main factors that influence the production of nanofiber by Forcespinning^TM^ are listed. Since it uses centrifugal forces instead of electric fields, the Forcespinning^TM^ technique provides distinguished advantages such as higher productivity rate, around 200 times higher than e-spinning, as well as a low-cost process and use of conductive and nonconductive polymer solutions [25].

### 2.3. Solution Blow Spinning

The solution blowing spinning or air-jet spinning is another non-electrical nanofiber spinning technique due it using compressed air as the driving force for the fabrication of nanofibers (40 nm). The main purpose of the creation of this technique is to overcome the limitation of e-spinning over in situ synthesis and conductive targets. It requires a conventional airbrush, a polymer solution, and a compressed gas source. The most common system used is based on parallel concentric nozzles, the inner nozzles contain a polymer solution and the outer nozzle has a pressurized gas flow. The polymer solution is driven out of the inner nozzle and stretched by a high-pressure stream of the compressed gas flow around the inner nozzle. The droplets are then formed into a conical shape, then when the air pressure exceeds the surface tension the droplets are stretched by the pressure force and the solvent is evaporated rapidly, solidifying the polymer and allowing its collection over the target of interest [26,27,28]. The main advantages of this system rely on its non-expensive costs, ease of transportability, and its feasibility making possible its application to any surface or substrate [26].

### 2.4. Carbon Dioxide (CO_2_) Laser Supersonic Drawing

CO_2_ laser supersonic drawing has been developed to produce nanofibers with the characteristic of having indefinite length due to the continuous supply of a laser beam of CO_2_ and a constant supersonic velocity [29]. In this technique, a supersonic jet is used by blowing air into a vacuum chamber through a cavity which is also used to supply the fiber into the vacuum chamber. While the fiber is entering through the cavity, the adiabatic expansion of air cools the jet and the fiber is melted by the CO_2_ laser that irradiates the cold [30]. Approximately, the nanofiber achieves diameters between 100 and 200 µm. Usually, this technique is employed in thermoplastic polymers such as polylactic acid (PLLA), polyethylene terephthalate (PET), poly(ethylene-2,6-naphtalene), polyglycolic acid (PGA), and ethylene tetrafluoroethylene (ETFE) [31,32,33,34]. The advantages that this technique offers are the absence of any solvent or additional processes to prepare the polymer and a higher yield than e-spinning [35].

Because of the extreme thermo-physical conditions applied during the solution blow spinning and carbon dioxide (CO_2_) laser supersonic drawing techniques, these are not used for the incorporation of herbal extracts into fibers due to degradation produced in these bioactive molecules.

## 3. Polymers in the Nanofiber’s Fabrication

**Figure 3:**
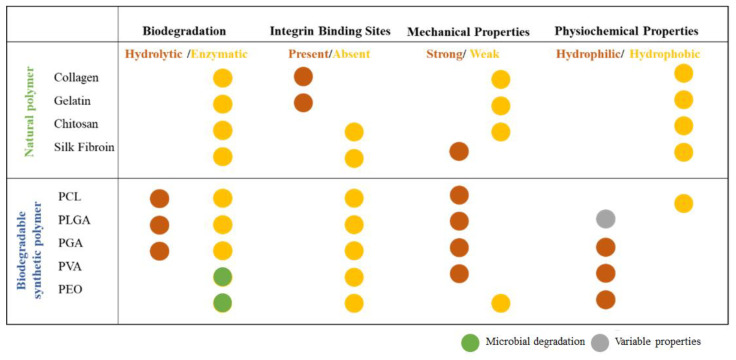
Physicochemical, mechanical, and biological properties of natural and synthetic polymers for nanofiber fabrication.

Several polymers, both synthetic and natural, have been used for healthcare applications since they are considered the most suitable class of materials due to their properties of biocompatibility and biodegradability. It is known that a biopolymer nanofiber scaffold would be an ideal material used in healthcare applications since it is enhanced with properties such as greater water retention capacity, it presents fewer immunogenic reactions, and its surface is soft to the touch, so it will not chafe the wounds or the area where it is applied [36] (Figure 3).

### 3.1. Natural Polymers

Natural biopolymers such as zein, silk fibroin, gelatin, keratin, collagen, and elastin have aroused special interest over synthetic polymers due their biocompatibility, biodegradability, hydrophobicity, non-carcinogenicity, and nontoxic effects in a biological system [37].

#### 3.1.1. Collagen

Collagen, a triple-stranded helical protein, is well known as the most abundant protein found in the human body (about 20–30% of total proteins) because it is the main component of the extracellular matrix (ECM) and the key component for the formation of tissues and organs [38]. Biologically, this protein has the function of structural support and regulation of cellular properties such as cell shape, proliferation, differentiation, and cell migration [39]. This protein has been widely used for the fabrication of e-spinning nanofibers due to its structural characteristics and protein properties such as biocompatibility, versatility, biodegradability, re-absorbability, synergy with other components, compatibility with other polymers, non-immunogenicity, and accessibility to its isolation from a bovine porcine or avian source [40]. Moreover, collagen has had great relevance because the formation of fibers can increase strength, transmit forces, dissipate energy, prevent early mechanical failure, and provide biological signals to adjacent cells [16]. 

Collagen nanofibers can provide the advantage of mimicking those nanofibrillar structures responsible for tissue integrity [41]. Collagen e-spinning nanofibers have faced the limitations of having poor mechanical properties and poor stability to aqueous solutions. The use of crosslinking methods such as the hybridization of collagen with other polymers has overcome those limitations and enhanced collagen properties for its clinical use. Some crosslinker reactants used are glutaraldehyde (GTA) for polycaprolactone (PCL), genipin for poly(lactic-co-glycolic acid) (PLGA), and EDC/NHS (1-Ethyl-3-(3-dimethylaminopropyl) carbodiimide/N-hydroxysuccinimide) for PLLA.

#### 3.1.2. Gelatin

Gelatin is a natural biopolymer found in tendon cartilage, connective tissues, and porcine or bovine bones derived from collagen hydrolysis by thermal denaturation or physical and chemical degradation. Depending on its pretreatment process, two types of gelatins are obtained: (i) type A or acidic pretreatment gelatin and (ii) type B or alkaline pretreatment gelatin. Acidic pretreatment can affect amide groups, whereas alkaline can hydrolyze amide groups of asparagine and glutamine to form aspartate and glutamate [42].

As well as fulfilling the characteristics of a natural polymer such as biocompatibility, biodegradability, hydrophobicity, non-carcinogenicity, and nontoxic effects in a biological system, gelatin is a low-cost material, possesses low antigenicity compared with native collagen, and displays integrin-binding sites for cell adhesion and differentiation [43,44,45]. 

By the e-spinning method, gelatin can be spun by using organic solvents and water, nevertheless, it has encountered some technical disadvantages due to the low volatility and high surface tension of water which prevents the formation of gelatin nanofibers as well as low conductivity, loss of polymeric solution viscosity, and impossibility to be spun at room temperature. Thus, solvents such as 1,1,1,3,3,3-hexafluoro-2-propanol (HFIP), 2,2,2- trifluoroethanol (TFE), and trifluoroacetic acid (TFA) have been used to enhance gelatin e-spinning nanofiber. Other authors have worked with the fabrication of gelatin nanofibers using less toxic organic solvents such as acetic acid and formic acid aqueous solutions [42,46]. According to Mamidi et al. [47], e-spinning limitations can be avoided by using the Forcespinning^TM^ method to obtain functional gelatin nanofibers with better mechanical properties, cell binding, and control over microarchitecture. 

However, gelatin nanofibers have faced the disadvantage of being dissolved in aqueous conditions. Gelatin has amine and carboxylic groups that can be ionized in water to get charged, so the polyelectrolytic properties and its strong hydrogen bonding make it impossible to obtain physically stable nanofibers [48]. To solve this, the crosslinking process and polymer mixtures have been used to improve water stability and thermo-mechanical properties of nanofibers [49,50].

#### 3.1.3. Chitin and Chitosan

Chitin (β(1–4)-poly-N-acetyl-D-glucosamine), the major component of invertebrates, crustaceans, mollusks, insects’ exoskeleton, and fungi’s cell walls, is the second most abundant natural polymer [51]. Recently, chitin has received special attention because it can be easily obtained from seafood industry waste, being considered as an eco-friendly polymer. Chitin is commonly converted to chitosan, its deacetylated derivative, either by enzymatic treatments or chemical hydrolysis. Deacetylation degree of chitin is an important characteristic to consider because it influences the chemical and physical properties of nanofibers such as solubility, flexibility, polymer conformation, viscosity, crystallinity, high surface area, mesh porosity, tensile strength, conductivity, and photoluminescence. On the other hand, it also influences biological properties such as biodegradability, biocompatibility, mucoadhesion, adsorption, antimicrobial activity, anticholesterolemic activity, and antioxidant activity [52]. Chitosan contains a repeating structure of 1,4-linked 2-amino-2-deoxy-β-D-glucans with free amino groups, which makes it a renewable, biodegradable, and promising biopolymer with unique properties, which could be improved by combining with other film-forming materials [17].

Chitin and chitosan have been used as a potential natural polymer because it is biocompatible, biodegradable, nonantigenic, and inexpensive. Moreover, this polymer offers a great opportunity for fabricating nanofibers since it has high mesh porosity, a predictable degradation rate, structural integrity, and nontoxic effects in cells [53]. 

Chitin is insoluble in organic solvents because of its crystalline structure and hydrogen bonding between carbonyl, hydroxyl, and acetamide groups [54]. On the other hand, the presence of primary amino groups in chitosan (pKa of 6.3) makes it soluble in dilute acidic solutions such as acetic, lactic, malic, formic acids, and water–methanol, water–ethanol, and acetones mixtures [55]. Little changes in pH levels (pH 6–6.5) can substantially alter charged state and chemical properties; for instance, at low pH, amino groups get protonated, convert positively charged, and therefore chitosan becomes a water-soluble cationic polymer. If the pH increases to above 6, the amine groups deprotonate, making the polymer insoluble [56]. Chitosan has also been blended with other polymers, such as poly(vinyl alcohol) (PVA), polyvinyl pyrrolidone (PVP), polyethylene-glycol (PEO) [57,58], poly (lactic-co-glycolic acid) (PLGA) [59], to improve its mechanical properties as well as coupled crosslinking agents such as UV radiation, genipin, hexamethylene, glutaraldehyde, and graphene oxide [60,61,62,63,64].

#### 3.1.4. Silk Fibroin

Silk fibroin, a protein produced from silkworm (*Bombyx mori*) cocoons, has been an interesting alternative for the production of nanofibers due to its characteristics of being biocompatible, biodegradable, morphologically flexible, adherable, and permeable [51,65]. Silk fibroin is mainly composed of two structural proteins known as fibroin heavy chain and light chain. Glu-like proteins known as sericin hold the chains together [66]. 

Silk fibroin heavy chain is amphiphilic and contains alternating hydrophobic and hydrophilic sequences. The hydrophobic sequences are highly conserved sequence repeats of Gly-Ala-Gly-Ala-Gly-Ser and less conserved of Gly-Ala-Gly-Ala-Gly-X (X being either Val or Tyr) and are responsible for silk fibroin crystalline regions (β-sheet plates), while hydrophilic sequences contain shorter and nonrepetitive structures (random coils and α-helix chains) responsible for amorphous regions [67,68]. Reactive side groups of the amino acid sequence such as amines, alcohols, phenols, carboxyl groups, and thiols allow chemical modifications in its structure in order to change silk fibroin hydrophilicity and charge. For example, carboxylic acid side groups have been derivatized with primary amines of peptides (RGD sequence) to improve cell adhesion while tyrosine residues have been modified to expand the range of functionalization [66]. 

For developing nanofibers for clinical purposes, sericin must be removed from silk fibroin by degumming in boiling alkaline reaction using sodium carbonate (Na_2_CO_3_) and then a regenerated fibroin solution is obtained. This is to prevent an immunologic reaction due to the detection of sericin as antigenic by T-cells [69]. The degumming process involves the disruption of the nanofibril structure affecting its mechanical properties. In this step, the concentration of Na_2_CO_3_ is a key parameter for the regeneration of silk fibroin. Babitha et al. [16] reported that concentrations around 0.05% and 0.005% only induce limited degradation of the protein chains so they can aggregate and form nanofibrils. On the other hand, a higher concentration of Na_2_CO_3_ can cause the formation of nanoparticles. 

After the degumming process, the fibroin fibers are dissolved in lithium bromide (LiBr) and then dialyzed with ultrapure water or polyethylene glycol to regenerate fibroin fibers [70].

Fibroin aqueous solutions are the most used to obtain silk fibroin nanofibers by electrospinning technique. However, the solvent systems used to dissolve degummed silk (CaCl_2_/water/ethanol, LiBr, NaCl) have also been used as electrospinning solutions. Previous studies revealed that using a polymer concentration of around 8–10%, an electric field of 5 kV, and 5–7 cm of distance is possible to obtain silk fibroin fibers with diameters lower than 40 nm. pH levels influence solution centration thereby affects the diameter of the nanofiber; lowering pH decreases the concentration while an increase in pH provokes gelatinization of the polymer [16]. To overcome mechanical strength and stability limitations of the regenerated fibroin crosslinkers such as solvent (alcohol) vapor, water vapor, genipin, or functionalized multi-walled carbon tubes or copolymers such as PEO, PEI, chitin, or cellulose acetate are used [71,72].

#### 3.1.5. Zein

Zein is a prolamine protein that comprises around 47% of corn endosperm. Typically, zein is known for being a low nutritional source because of its deficiency in essential amino acids such as tryptophan and lysine, as well as for being an alcohol-soluble protein [73,74,75]. Recently, investigations have been carried out to overcome these limitations, giving another use of this protein by considering it as a biopolymer instead of a food residue [75]. Today, this protein has a special interest in being used as an alternative to produce nanofibers for clinical purposes due to its characteristics of being biocompatible, renewable, nontoxic, resistant to microbial degradation, and biodegradable since it comes from a natural source [76].

Zein nanofibers have been developed by e-spinning through the use of alcohols such as aqueous methanol and ethanol and other solvents such as acetic acid, formic acid, and dimethylformamide (DMF) [16]. To improve the physicochemical characteristic of zein nanofibers, this protein has been coupled with other polymers or treated with crosslinking processes, so its stability in solvent solutions and mechanical strength increase. Polymers such as chitosan, cellulose gelatin, tannin, polycaprolactone (PCL), collagen, and silk fibroin have been used for hybrid electrospinning, while reactants such as glutaraldehyde (GTA) and hexamethylene diisocyanate (HDMI) have been used for the crosslinking process. 

More recently, Deng et al. [77] have reported the use of glucose as a crosslinker via Maillard reaction to improve hybrid gelatin/zein nanofibers and avoid the use of chemical reaction crosslinking. According to this investigation, by using glucose, the mechanical properties of the nanofibers are enhanced and the conformation and interaction due to the reduction of sugars create nanofibers with tunable properties. In addition, the use of glucose improves cell viability and proliferation using crosslinked gelatin/zein nanofibers at different ratios.

#### 3.1.6. Soy Protein

As another alternative to animal-derived protein-based polymers, soy protein has been used for the fabrication of nanofibers. This plant protein is considered a renewable, nontoxic, biodegradable, biocompatible, and cost-effective polymer [78]. Additionally, this polymer has presented advantages for nanofiber fabrication such as high polarity to improve cell attachment, presence of isoflavones (genistein and daidzein), and bioactive peptides similar to extracellular matrix proteins that can promote adhesion, proliferation, and migration of cells [79,80].

Soybeans contain around 38% protein, 30% carbohydrates, 18% oil, and 14% moisture and minerals [80]. Three different products from soybean are obtained: soy flour (SF), soy protein concentrate (SPC), and soy protein isolate (SPI). Among them, SPI has been widely used for the development of nanofibers due to its high content of protein (around 90%) [81].

Soy protein is abundant in amino acids such as aspartic and glutamic acid, alanine, valine, leucine, lysine, arginine, cysteine, and glycine [80]. The high number of reactive groups (-NH2, -OH, -SH) allows chemical, physical, and enzymatic modifications of the protein [82]. In contrast to other natural polymers which can be easily dissolved and directly used to create nanofibers, the globular structure of the soy protein must be unfolded by denaturalization before being spun. The most common technique is the disruption of the quaternary structure by an alkaline-thermal treatment [83]. By exposing hydrophobic and sulfhydryl groups, the protein unfolds and solubilizes at a pH above the isoelectric point (pH 4.5), so these groups can form hydrophobic and disulfide bonds to stabilize the protein link. In addition, by heating the protein, soybean tissue is softened, the quaternary structure is denaturalized, and harmful substances (trypsin inhibitor and hemagglutinin) are inhibited [84]. 

SPI nanofibers have been fabricated by using water, hexafluoroisopropanol (HFIP), trifluoroethanol (TFE), Triton X1000, and NaOH as solvents. Moreover, due to its low solubility and low mechanical strength, SPI nanofibers have been improved by blending SPI with other polymers and using crosslinker solutions [16]. Soy protein reactive groups allow that the protein can chemically or physically be joined with other polymers such as PEO, PLA, PVA, or PCL. In addition, because of the presence of reactive groups, SPI can be crosslinked by a cross-linker solution (formaldehyde, glutaraldehyde, glyoxal), heat treatment, enzymatic treatment, or irradiation. The most used are formaldehyde and glutaraldehyde, which form intra and intermolecular cross-links by reacting first with the free amine groups of arginine, lysine, and hydroxylysine residues. Nevertheless, these cross-linkers have aroused concerns about its cytotoxic effect, glyoxal being a less toxic alternative [85].

#### 3.1.7. Cellulose

Cellulose, the most abundant organic polymer, represents about 1.5 × 10^12^ tons of the planet’s total biomass production per year. Currently, this polymer is considered an unlimited source of raw material to overcome the high demand for eco-friendly products. Its properties such as biodegradability (microbial degradation), biocompatibility, availability as a renewable source, scalability, and profitability have allowed its use as a potential alternative polymer for the fabrication of nanofibers [86]. Native cellulose is typically found as highly fibrillated and semi crystalline, making this polysaccharide difficult to dissolve in organic solvents, recalcitrant to be converted to small molecules, and with the predisposition to aggregate or form gel, affecting its use for nanofiber fabrication [87]. 

Cellulose electrospinning nanofibers have been developed by dissolving it using N-methyl morpholine-N-oxide/water (NMMO/H_2_O), lithium chloride/N and N-dimethylacetamide (LiCl/DMAc), and ionic liquids such as 1-allyl-3-methylimidazolium chloride (AMIMCl)/dimethylsulfoxide (DMSO). Nevertheless, due to the high melting temperature of NMMO, the cellulose/NMMO hydrate solution needs to be processed at temperatures around 80–130, resulting in less uniform nanofibers which have to be washed in distilled water to remove the residual solvent [88]. Furthermore, in the case of AMIMCl, residual ions have to be removed by coagulation in ethanol, the solvent from the cellulose nanofibers being difficult to eliminate [87]. 

In order to facilitate the fabrication of cellulose nanofibers, the use of cellulose derivatives such as cellulose acetate, hydroxypropyl cellulose, hydroxypropyl methyl cellulose, and ethyl-cyanoethyl cellulose has been proposed instead of direct cellulose [89]. Among them, cellulose acetate (CA) was the first option to develop electrospinning nanofibers because it is biodegradable, biocompatible, insoluble in water, with good mechanical properties, and hydrolytic stability, nontoxic, chemical resistant, and a low-cost material. CA can be generated by acetylation of hydroxyl groups in cellulose, ring-opening esterification, iminium, chloride, N, N-carbonyl diimidazole, and transesterification [86].

In comparison with cellulose, CA is soluble in common organic solvents such as acetic acid, acetone, chloroform, dimethylformamide (DMF), dimethylacetamide (DMAc), dichloromethane (DCM), formic acid, and pyridine [88,90]. Mixed solvents systems of DMAc with acetone or acetic acid have enabled the production of more efficient CA nanofibers. One advantage of using CA instead of other cellulose derivatives is its capability of being reverted to cellulose by deacetylation processes using either NaOH or KOH. Therefore, the resultant nanofiber can be a cellulose nanofiber with better mechanical properties and production rates [91,92]. CA nanofibers have also been improved by the incorporation of other polymers such as PEO, PCL, and PHB [93,94,95,96].

### 3.2. Biocompatible Synthetic Polymers

Biocompatible synthetic polymers such as poly (ε-caprolactone) (PCL), polyglycolide (PGA), polylactic acid (PLA) and its copolymers, poly (lactic-co-glycolic acid) (PLGA), poly(vinyl alcohol) (PVA), and polyethylene-glycol (PEO) are commonly used to improve natural polymer nanofibers properties such as hydrophilicity, chemical, and thermal stability [97].

Furthermore, biocompatible synthetic polymers enhance mechanical properties and degradation kinetics, and allow to set specific morphologic features. Table 2 lists the main biocompatible polymers used in nanofiber systems. Some of them are considered biodegradable polymers too, such as the polyesters PLA, PGA PLGA, and PCL. Biodegradability allows bond cleavage of the polymer hydrolytically or enzymatically to polymer degradation. Synthetic polymers are commonly hydrolytically degraded since they are considered biologically inert. Among them, polyesters confer and ease degradation ability by hydrolysis of ester linkage so degradation products can be absorbed through the organism by metabolic pathways. 

## 4. Incorporation of Active Compounds

Given the beneficial properties of bioactive compounds, there has been an increasing interest in incorporating them into several delivery systems, since they sometimes present low bioavailability or are needed to have a more controlled release on different surfaces. The incorporation of active compounds into appropriate carriers may enhance their properties due to characteristics such as higher surface area and enhanced mechanical properties [105].

### Natural Extracts

The use of natural extracts or herb bioactive compounds from plants has gained a special interest in current clinical technology, even though they have been used since ancient times. This is due to the beneficial properties and the healing performance of their secondary metabolites for the treatment of diseases, as well as for being a nontoxic and potentially biocompatible pharmacological alternative that can reduce side effects compared with synthetic drugs. These natural extracts are considered resources of new drugs due to the notable diversity of chemical structure and biological activities given by their constituents [106]. 

Several plants have shown therapeutic effects; due to this, they have been incorporated into delivery systems to enhance properties such as stability and liberation, among others (Table 3). Plant bioactive compounds are the key factors for the extract’s therapeutic properties such as hypoglycemic, antidiabetic, antioxidant, antimicrobial, anti-inflammatory, and anticarcinogenic, among others. Süntar [107] points out that by analyzing and using natural extracts from plants as medicine, it is possible to achieve different clinical therapeutic goals such as isolating bioactive compounds for direct use as a drug, synthesizing novel chemical structures from a known bioactive compound, and use of bioactive compounds as pharmacological tools. 

The main bioactive compounds currently studied for the development of herbal-derived drugs are saponins, tannins, flavonoids, alkaloids, and quinones [125]. Most of these compounds are known to be highly soluble in water but they are unable to cross lipid membranes, have a high molecular size, and have inefficient absorption rates converting them as poor bioavailable and non-effective therapeutic agents. As a solution, nanotechnology has enabled the development of nanostructured systems to potentiate the action of bioactive compounds [126]. Due to its characteristics, such as high mesh porosity, nanofiber systems can have a larger surface area, allowing molecules to diffuse out from the system gradually to the desired site of action.

Some authors have studied different plants rich in phenolic compounds such as *Lawsonia inermis L(Henna)* [127], *Centella asiatica* [128], *Chamomilla recutita* (L.) [129], and *Stryphnodendron adstringens* [130] to validate its incorporation into fiber production. For instance, Hani et al. [131] coupled *Moringa oleifera* leaf crude ethanolic extract rich in quercetin, kaempferol, and ascorbic acid with gelatin to generate a nanofiber via the e-spinning method. This is with the purpose of providing an alternative matrix with the capacity of acting as a potential oxidative stress inhibitor. 

Moreover, *Herba epidermii* has been used to develop a PCL/gelatin microfiber loaded with a flavonoid glycoside, icariin (ICA), as an efficient release system. Due to ICA allowing the inhibition of collagen and fibronectin accumulation and having anti-angiogenic activity, ICA PCL/gelatin microfibers were studied to validate its use as a system for the prevention of scar and adhesion formation. In vitro studies were performed to assess cell attachment and microfibers’ barrier function to fibroblast as well as in vivo studies to evaluate its degradation and degree of adhesion in tissue. According to the results, by using ICA-PCL/gelatin microfibers, fibroblasts cells had a normal growth on the frontal membrane and no cell infiltration to the opposite side or adhesion at the bottom demonstrating its good barrier functionality. In addition, fibroblast adhesion and viability decrease gradually, confirming its use as an anti-adhesion matrix [132].

## 5. Applications in Healthcare

### 5.1. Wound Dressing

A wound is defined as the disruption to the normal anatomical structure and function. It can occur from a simple breakage of the epithelial integrity of the skin to a deeper disruption that extends into subcutaneous tissue affecting other structures (tendons, muscles, vessels, nerves, parenchymal organs, and bone). Wound healing is achieved by four main steps: (1) hemostasis, (2) inflammation, (3) proliferation, and (4) remodeling. The whole process involves complex and dynamic coordinated mechanisms such as bleeding, coagulation, initiation of inflammation, regeneration, migration, and proliferation of connective tissue and parenchyma cells, synthesis of ECM proteins, and remodeling of the new tissue [133,134]. 

Considering this, a wound dressing is vital for the management of thermal, chronic, and traumatic wounds and for the regeneration of epidermal and dermal tissues. An ideal wound dressing must accomplish different characteristics such as maintaining a moist environment at affected tissue, absorbing the excess of exudates, being non-adherent, nontoxic, non-allergenic, and easy to manipulate. Moreover, it must provide a debridement action, give mechanical stability to allow cell growth, achieve rapid hemostasis, act as an antimicrobial barrier, prevent cellular dehydration, promote collagen synthesis and angiogenesis, stimulate the growth factors, and allow gaseous exchange and to be porous so it can allow diffusion of wastes and nutrients. Traditionally, materials such as gauze, plasters, bandages, cotton, and wool are used for wound protection, but they encounter some limitations such as adhesion to the wounds, wound dryness, and lack proper absorption of exudates [125]. Recently, modern technologies have enabled the enhancement of these conventional wound healing systems to produce commercial wound dressing systems.

Another technology to achieve the ideal characteristics of a wound dressing are nanofiber systems. They can possess similar architectural features and morphologies to the ECM and due to their large surface and high mesh porosity, they allow the adhesion and proliferation of epidermal and dermal cells such as the secretion of collagen and growth factors for the ECM remodeling and reconstruction of the affected tissue [135].

On the other hand, nanofibers can couple with bioactive compounds to create active or multifunctional wound dressings. Wound healing can be accelerated, and skin recuperation enhanced by the biological activities of bioactive molecules. Natural extracts from plants can nurture the wound site due it possessing emollient, demulcent, epithelializing, astringent, antimicrobial, anti-inflammatory, and antioxidant properties. Furthermore, with this purpose, natural polymers such as collagen, chitosan, and soy protein, among others, have been used due to their biocompatibility of substrates commonly recognized by the organism. However, they are frequently improved by hybridization with synthetic polymers or crosslinking agents to increase their mechanical properties [51]. 

**Figure 4:**
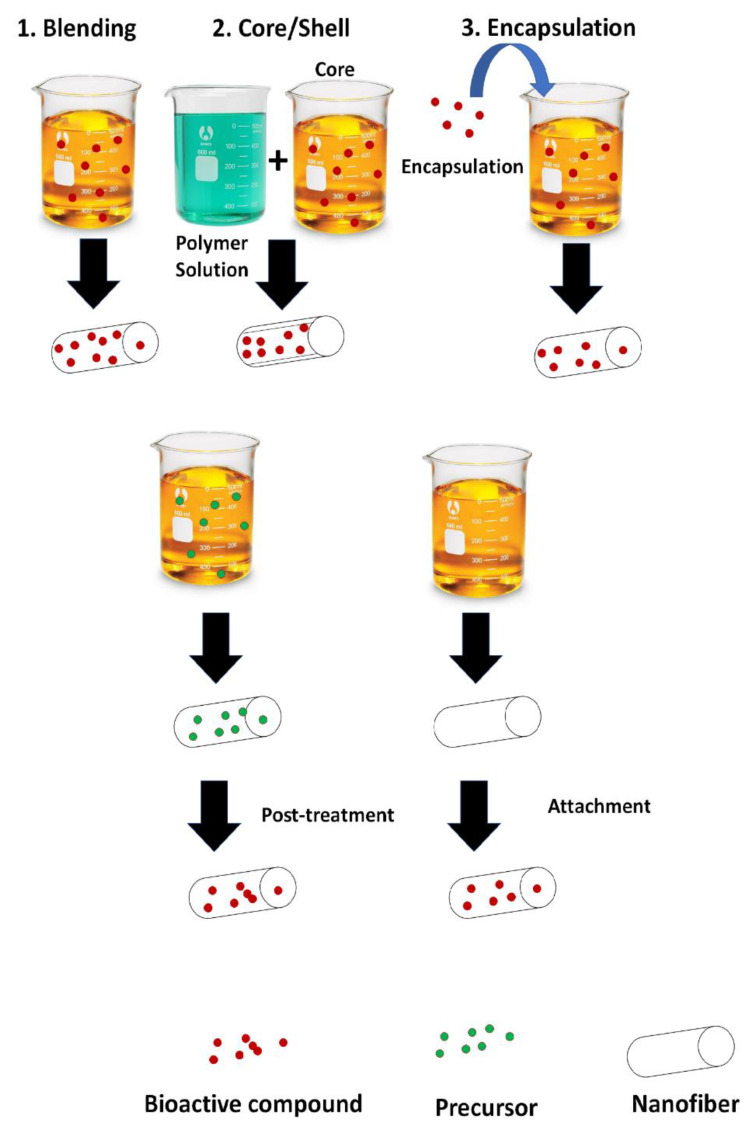
Methods for incorporation of bioactive compounds to nanofibers.

Active nanofibers are usually manufactured by the incorporation of those bioactive compounds of interest with the selected polymers. Different methods are commonly used to achieve this combination such as: (i) blending the active compound with the polymer before spinning, (ii) fabrication of a core/shell structure by coaxial spinning, (iii) encapsulation of the bioactive compounds before blending with the polymeric solution, (iv) post-treatment of the nanofiber, and (v) attachment of the bioactive compounds to the nanofiber’s surface (Figure 4) [136].

Adeli-Sardou et al. [127] analyzed the incorporation of lawsone in wound healing systems. In this investigation, different concentrations of lawsone in a solution of PCL and gelatin were electrospun. PCL-gelatin-lawsone nanofiber increased HGF cells growth and proliferation, presented antibacterial activity against *S. aureus*, and demonstrated by in vivo assays its wound closure potential by promoting a wound contraction of 96.3 ± 4% and 100%. Another study fabricated *Lawsonia inermis* extract coupled with gelatin-oxidized starch (OST). Oxidized starch has the ability to easily react with the amino groups of gelatin and crosslinked gelatin. This matrix enhances the attachment and proliferation of fibroblast cells, collagen secretion, and demonstrated antibacterial activity. Gelatin-OST-extract nanofibers were assessed in vivo on a second-degree partial burn wound, demonstrating a thinner inflammatory zone as well as less inflammatory response and macrophage amount compared with those control fibers [137].

Chitosan-based nanofibers have also been assessed to design novel wound dressing systems incorporated with bioactive compounds. *Garcinia mangostana* extracts have been incorporated into a chitosan-ethylenediaminetetraacetic acid-polyvinyl alcohol nanofiber (CS-EDTA-PVA) showing antibacterial against *S. aureus* and *E. coli* strains and antioxidant activity. They also showed cell viability in NHF above 90% using GM extract concentrations between 1 and 5 mg/mL and accelerated the wound healing process compared with traditional and commercial gauze dressing [138].

Yang et al. [139] worked with silk fibroin-based wound dressing incorporated with manuka honey (MH). This investigation demonstrated antimicrobial activity dependent on MH concentration against Gram-negative and Gram-positive bactera (*E. coli*, *P. aeruginosa*, *S. aureus* and methicillin-resistant *Staphylococcus aureus (MRSA)*). Silk fibroin-MH nanofibers also showed an increase in wound healing by stimulating the release of cytokines and inducing an immune response against skin infection. Ranjbar-Mohammadi et al. [140] reported electrospun curcumin-loaded PCL/gum tragacanth (GT) (PCL-GT-Cur) nanofibers as an antimicrobial and wound healing dressing for diabetic wound treatment. Results determined the antibacterial activity of 99.9% against MRSA and 85.14% against ESBL. In in vivo assays, PCL-GT-Cur nanofibers increased collagen content, accelerated the healing process, and decreased blood glucose levels in diabetic rats. Mishra et al. [141] also reported the fabrication of antibacterial lemongrass oil-loaded composites of cellulose nanofibers (CNFs)-polyethylene glycol (PEG). Nanofibers showed antimicrobial activity against *S. aureus* and *E. coli*.

Another work showed the fabrication of soy protein hydrolysate (SPH)/cellulose acetate (CA) nanofibers by rotary jet spinning to enhance skin regeneration. Soy protein can mimic the fibrous dermal ECM microenvironment; due to this similarity, soy peptides can promote cell adhesion, proliferation, and migration. In addition, since it carries phytoestrogens, they can bind to specific estrogen receptors which have positive effects on ER-mediated processes to accelerate the healing process. Coupled with this, soy peptides are reported to have antibacterial, anti-inflammatory, and antioxidant properties. SPH/CA nanofibers showed low cytotoxicity in human dermal fibroblasts (HNDF) and an increased integrin β1 expression indicating that the nanofiber can activate the expression of integrin β1 and therefore accelerate cell migration and start the production and maturation of ECM proteins. In vivo assays in mice demonstrate accelerated wound closure and skin regeneration [79]. Yet another study reported the incorporation of plant extracts from *Indigofera aspalathoides, Azadirachta indica, Memecylon edule* (ME), and *Myristica andamanica* with a biodegradable polymer, PCL by electrospinning. The authors describe a positive proliferation of human dermal fibroblast (HDF) and high cell viability in presence of extract-PCL nanofiber [3].

### 5.2. Tissue Engineering

**Figure 5:**
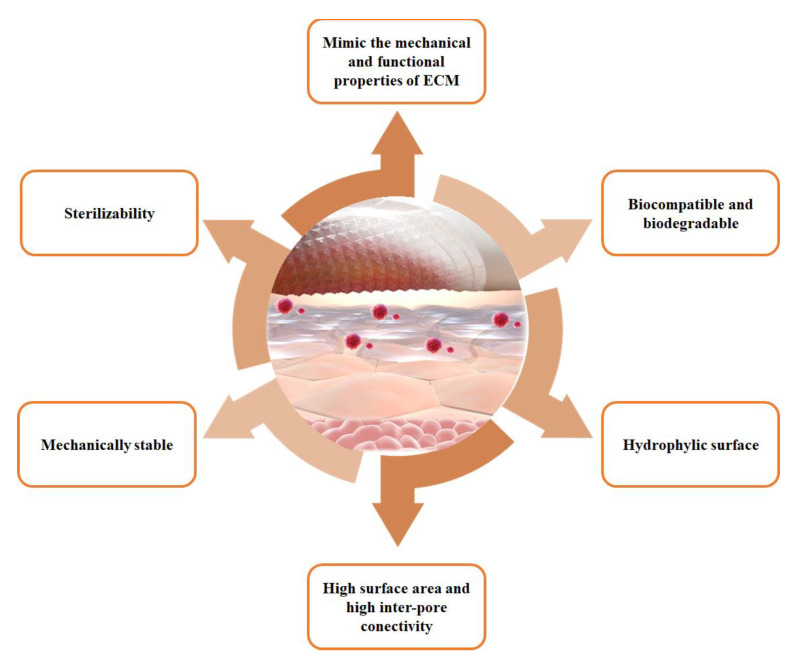
Main characteristics for tissue scaffold design.

Commonly, when there is a tissue injury or organ failure, organ transplantation of the organ or tissue is the first clinical option to restore the functionality of the affected area. Nevertheless, this method leads to certain biological limitations such as the allorecognition, graft rejection, and administration of immunosuppressive agents, among others. As a solution, tissue engineering has aroused special attention as an “interdisciplinary field that applies the principles of engineering and life sciences toward the development of biological substitutes that restore, maintain, or improve tissue function” [142]. Tissue engineering strategies have to consider three aspects: (1) biomaterial scaffold production, (2) cell seeding and attachment, and (3) incorporation of cellular signaling factors. Among them, the most important factor is scaffold production. The scaffold may accomplish different characteristics to achieve the ultimate purpose of tissue engineering: develop a functional matrix that resembles the structural architecture of the native tissue to enhance resynthesize of new natural ECM (Figure 5) [134].

ECM proteins provide structural support and integrity to tissues, promote cell adhesion, and regulate cellular mechanisms. Differences in ECM structure and organization may be attributable, in part, to variation in size and hemodynamic burden of its different components. Thus, nanofibers are a suitable option to resemble native ECM because they are at the same scale as ECM proteins. Furthermore, its properties such as mechanical stability, high specific surface area, mesh porosity, biocompatibility, and biodegradability allow its usage as a scaffold [143]. Natural polymers such as agarose, chitosan, collagen, gelatin hyaluronan, alginate, and bone matrix are frequently used because of their biocompatibility and functional motifs. However, natural polymers are coupled with synthetic polymers such as PCL, PLLA, and PLGA to provide stability, mechanical strength, and bioactivity [41]. Furthermore, herbal extracts can be incorporated into these nanofibrous scaffolds to provide additional functionality owing to their therapeutic effects for disease treatment. Nemati et al. [144] described nanofibers as versatile systems since they can act as a drug delivery system as well as a scaffold to alleviate clinical symptoms by the controlled release of a specific bioactive compound, preventing microbial infections, and promote tissue regeneration.

Selvaraj and Fathima [145] reported a fenugreek/silk fibroin skin scaffold fabricated through co-electrospinning using HFIP. Fenugreek from *Trigonella foenum graecum* possesses antioxidant properties which allow to reduce wound damage by reducing reactive oxygen species during inflammation, while silk fibroin promotes collagen synthesis and proliferation of epithelial cells. Results show a cumulative release of fenugreek of 73 ± 0.9%. Fenugreek presence in silk fibroin nanofiber confirms its antioxidant property, by obtaining 49.3% of antioxidant property through 2,2-diphenyl-1-picrilhidrazil (DPPH) assays. Cell viability assays with albino 3T6 fibroblast showed 100% viability while cellular behavior analysis showed an increase in cell proliferation and adhesion to the scaffolds. Moreover, fenugreek/silk fibroin nanofibers obtain higher wound closure rate (95.8%) compared with gauze and silk fibroin nanofiber (78.9% and 93.7%).

Another study fabricated curcumin/zein nanofibers by electrospinning nanofibers optimizing zein nanofibers with fluorescence to observe curcumin incorporation. The addition of curcumin potentiates formation of granulation tissue, ECM biosynthesis, cell proliferation, and promotes antioxidant properties. Results showed an enhanced attachment and proliferation of L929 fibroblast cells, suggesting the cytocompatibility of the nanofibers. Furthermore, antioxidant activity was dependent on curcumin concentration in nanofibers, obtaining a 91.2% inhibition using the highest concentration (10 wt%) [146]. 

*Aloe vera* (AV) gel has also been incorporated into PLLA/collagen nanofibrous scaffold by electrospun. The authors described the use of *Aloe vera* gel, since it can accelerate the wound heling process due to the presence of glycoproteins, saccharides, vitamins, and antioxidants. PLLA/collagen/AV scaffolds promote a favorable environment for cell survival, proliferation, and adhesion of L929 fibroblasts [147]. Recently, Shahbazi and Bahrami [148] described the fabrication of honey nanofibers coupled with antimicrobial herbal extracts from *Allium sativum* L., *Mentha spicata* L., and *Agaricus bisporus* for their use as skin and bone scaffolds.

### 5.3. Drug Delivery

In recent years, nanotechnology has enabled the development of new drug delivery systems which contain a specific amount of a bioactive compound and control the rate and time of release at a specific site of the organism. Nanofibers have roused special interest as a drug carrier system for being a matrix with high surface area and mesh porosity. According to Thakkar and Mirsa [149], the drug release performance can be known considering the physical characteristics. For instance, if the diameter is smaller, the release rate will be faster due to the high surface area and dissolution rate. Moreover, mesh porosity can affect release speed; thicker fibers with high mesh porosity can release the compound faster than thinner fibers with low mesh porosity. Likewise, alignment of the nanofiber can affect compound release: randomized patterns are reported to have faster release due to an increased predisposition of water uptake. The main physical characteristics of nanofiber for drug release are (i) alignment, where the more randomized, the faster bioactive compound release, (ii) diameter, where thicker nanofibers promote the slower rate of release, (iii) crystallinity, where high crystallinity polymers promote the slower release, compared to amorphous polymers, (iv) porosity, where the higher the mesh porosity, the faster the release rate. 

Other parameters are those related to polymer characteristics such as composition, crystallinity, and molecular weight. For example, hydrophilic and amphiphilic copolymers can increase compound loading and decrease probabilities of burst release, while polymers with amorphous regions can have faster release rates unlike those with crystalline regions, in which water uptake is lower. Finally, bioactive compound loading parameters, such as loading amount, molecular weight, physical state, solubility, and compound-polymer interaction, can also influence the release performance of the nanofibers [150].

Depending on the final drug release characteristics, six techniques have been reported for the attachment of the desired drug with a nanofiber: (1) co-spinning, (2) side-by-side-spinning, (3) multi jet, (4) coaxial, (5) emulsion spinning, and (6) surface immobilization. Among them, co-spinning is the most used since the drug and polymer can be blended before proceeding with the nanofiber method. Nevertheless, some limitations are encountered by this loading method. First, the bioactive compounds may be compromised using high voltages conditions or method temperatures. Second, incorporation of both bioactive compounds and polymers may be tough if they are not soluble in the same solvent. To overcome these limitations, loading methods such as side-by-side and multi jet can be used, since polymer and bioactive compounds solutions can be added separately. In addition, core/shell structure nanofibers can be fabricated using coaxial and emulsion spinning, while surface immobilization involves covalent coupling of bioactive compounds to the surface of the nanofiber [136,151].

The incorporation of natural extracts or herbal bioactive compounds has been studied to create a sustainable and accurate release system of a herbal drug. Nanofibers can promote the activity of herbal bioactive compounds, reduce the effective dose, reduce side effects, and guide the final action site. One key factor to develop these release systems is to consider the chemical complexity of the bioactive compound to ensure the active form of the compound when it is released. Nanofibers could improve the solubility of the drug and bioavailability, improve compound stability, and reduce physical or chemical degradation rate and cytotoxicity such as control absorption rate of herbal extracts [152]. 

Sedghi et al. [62] reported the fabrication of chitosan (CS)/PVA nanofiber loaded with curcumin (CUR). In this study, PVA-CS was produced by the electrospinning and crosslinked using 3-aminoprpopyltriehoxysilane (APTES) modified graphene oxide in Si-O-Si networks for drug entrapment. fGO-Si-CUR nanofibers show a reduction in the initial burst of the bioactive compound (37%), in comparison to non-crosslinked nanofibers (57%); demonstrating their ability to sustain and prolong the release of the drug. In addition, cytotoxicity was assessed to observe its potential as an anticancer drug delivery system using MCF-7, HepG2, and L929 cells lines. fGO-Si-CUR nanofibers show an effective inhibition of cell growth of 80% for MCF-7, 100% for HepG2 and L929. Antibacterial assays were accomplished by evaluating the inhibition of MRSA and *S. epidermis*, obtaining inhibition percentages of 94.24 ± 1.69% and 88.42 ± 2.81%, respectively. The authors mention the use of these nanofibers as a postoperative chemotherapy treatment or antimicrobial therapy. 

Cellulose acetate nanofibers loaded with vitamins were fabricated by Taepaiboon et al. [153] for transdermal application. Vitamin E and Retin A were loaded by co-electrospinning to the CA solution using acetone/N, N-dimethylacetamide as solvents. This study analyzed the release activity of the nanofibers and vitamins stability. Retin-A showed lower stability than vitamin E after the electrospinning process, due to its loss of chemical characteristics in high voltage conditions. About its release stability, Vitamin E showed stability in B/T/M medium (0.5 vol% Tween 80 and 10 vol% methanol in acetate buffer solution) for 24 h and Retin A for 6h and presented a gradual cumulative release of 52% and 34%, respectively. 

Other authors have encapsulated vitamin A in different concentrations in cress seed mucilage/PVA nanofibers to increase vitamin A solubility in aqueous media and decrease the vitamin degradation rate. Results show an encapsulation efficiency of 97%. Release activity was evaluated by simulated gastric and intestinal fluid, only 5.27% of the vitamin was released in gastric fluid [154]. Tavassoli-Kafrani et al. [155] reported the fabrication of gelatin and gelatin-crosslinked tannic acid nanofibers loaded with orange essential oil obtaining encapsulation efficiency of 69% and 52.6%.

Chantarodsakun et al. [156] reported the fabrication of [6]-Gingerol loaded cellulose acetate nanofibers. Gingerol, extracted from ginger, is known for showing immunomodulatory, anti-tumorigenic, anti-inflammatory, anti-apoptotic, antihyperglycemic, anti-emetic, and analgesic properties. The authors found 92% of release effectivity of the bioactive compound using acetate buffer solution. The results obtained also show the antioxidant activity of the nanofibers and viability of 65% in L929 cell line. Zhu et al. [157] prepared Asiaticoside-loaded alginate/PVA/Chitosan nanofiber by coaxial electrospinning. Asiaticoside, derived from *Centella asiatica* (L.), possesses anti-inflammatory and the antioxidant activities as well as antidepressant-like effects. The Asiatcoside alginate/PVA/chitosan presented higher release activity than a commercial herbal cream. In addition, nanofibers activate factors for adhesion and proliferation of keratinocytes and fibroblast, collagen secretion and extracellular matrix remodeling such as VEGF, CD31, PCNA, IL-6, and TNF-α.

### 5.4. Food Packaging

One application that has provoked interest among both the scientific community and the consumer demands has been the incorporation of natural biopolymer ingredients for food packaging, which addresses environmental concerns by reducing the use of petroleum-based plastic packaging [158].

Some of the most used polymers used in food packaging are chitosan and gelatin since their chemical composition allows them to have excellent inherent properties such as degradability, edibility, film-forming property, and compatible nature. Specifically, composites made by electrospinning of both materials have been reported to have enhanced properties suitable for this application such as food protection, promoting transportation, guaranteeing hygiene, and prolonging the shelf life of perishable items like those prone to stress, air, light, or even temperature [135]. 

An example of this is reported by Leena et al. [123] with the incorporation of commercial resveratrol via nanoencapsulation with zein using the electrospinning technique for its application as carriers of bioactive compounds. This enhanced the bioavailability and bioactivity of resveratrol *in vitro*, proving its potential as thin edible coatings with good barrier properties, controlled moisture loss, and color retention that could be used in nutraceutical oral delivery applications.

On the other hand, the incorporation of *Momordica charantia* fruit extract to coaxial shell core zein/gelatin electrospun nanofibers in order to enhance its antioxidative content and potency has been reported due to its content of flavonoids and polyphenols. This method has proven to deliver high encapsulation efficiency and sufficient shelf stability, highlighting its potential as a stand-alone nutraceutical supplement or as an ingredient for fillers or edible wrappers in various food products [124].

Another example of this application is the use of gelatin, chitosan, and PLA antibacterial nanofiber films elaborated by electrospinning. This method produced continuous, uniform nanofibers with improved thermal ability, moisture content, water-solubility, water vapor permeability, hydrophobic properties, and with a strong bactericidal effect in a short time, proving they are a safe, non-toxic, degradable option that ensured the safety, and quality of food products, increasing their shelf life [159].

### 5.5. In Vivo Studies

As previously mentioned, there has been a wide discussion about the general characteristics of drafts for wound healing, both mechanical and biological properties and function; nevertheless, just a few have been able to take their investigations beyond laboratory work to pre-clinic assays in animal models to ensure biological performance and toxicity effect in vivo.

Some of these researchers have focused on the nanofiber’s ability to improve wound healing. For example, Mahmoudi, et al. [160] created temporary skin grafts based in chitosan-genepin-graphene oxide electrospun nanosheets, where the open wounds were fully regenerated after 14 days with no visible scars. Similar to this, Uppal et al. [161] made hyaluronic acid (HA) nanofibers, showing the previously mentioned desirable mechanical properties (average thickness of 0.041 cm, 20.6% of degree of crystallinity, high air permeability) and, compared to other five types of dressings, the HA nanofibers showed the best results for wound closure. Additionally to these, alginate-based electrospun nanofibrous mats were produced by cross-linking with PVA and, in comparison with some other non-nanofibrous mats, the electrospun mat showed the best healing potential [162]. Another work studied the therapeutic effects of silk fibroin/gelatin (SF/GT) electrospun nanofiber dressing loaded with astragaloside IV (AS) on acute trauma, showing that the AS-loaded SF/GT nanofiber dressing group had a significantly higher wound healing closure rate compared to the control group, proving its healing accelerating ability and anti-scarring effect [163]. Similar to this, a bio-nanofiber of chitosan/PVA incorporating honey and *Nepeta dschuparensis* plant was produced (uniaxially aligned, sizes from 95–150 nm), revealing that after 21 days the wound healed faster by the incorporation of honey and *Nepeta dschuparensis* plant into the nanofiber mats [164]. Moreover, the wound healing effect of novel *Lihospermi radix* (LR) extract-containing bilayer scaffold was examined, where the porous morphology and high swelling ratio provided efficient exudate absorption ability with optimal characteristics for cell attachment and skin tissue regeneration [109]. These in vivo studies on rat skin showed an accelerated healing effect in large open wounds, promoting the healing process as the wound closure rate was significantly enhanced. 

On the other hand, some other in vivo studies have researched other properties that promote healthcare applications. For example, a scaffold was made by electrospun chitosan microfibers to function as a support for attachment and viability of rat muscle-derived stem cells (rMDSCs), showing adequate 3D structure and porosity allowing sufficient cell seeding conditions to facilitate cell proliferation and differentiation by allowing the transport of nutrients and oxygen into and out the scaffold, observing new blood vessels and presence of macrophages, Kang et al. [165]. Additionally, the anti-inflammatory activity of electrospun alginate micro/nanofibrous dressings loaded with the aqueous extract of *Pinus halepensis* bark (PHBE) was studied, showing that topical application of said patches on UV-inflamed skin significantly attenuated inflammation damage, reducing the healing period [166]. Moreover, pomegranate, Manuka honey, and bee venom, in combination with PVA nanofibers were fabricated for use as antibacterial wound dressings, showing moderate swelling and higher weight loss capacities were detected when compared to PVA mats, as well as significant activity against *S. aureus* and *E. coli* [167]. These in vivo studies proved that natural extracts incorporated into nanofibers could be used as a biocompatible scaffold, concluding that these nanofibers are promising for wound healing.

### 5.6. Commercially Available Scaffolds

According to StatNano.com database [168], currently in the market, there are about 60 products with nanotechnology focused on tissue engineering. All of these products are manufactured from about 25 companies in 10 countries worldwide, with the USA leading with 36 products, Japan with 7, Sweden with 6, and other countries with 2 or fewer products: Turkey (2), Czech Republic (2), Belgium (1), Hungary (1), Canada (1), and Spain (1). The main types of products available in the market are scaffolds (45), followed by antibodies, antiangiogenesis, hydrogels, and others. Finally, the properties that these products promise are biocompatibility, biodegradability, anti-microbial activity, collagen production, anti-inflammatory activity, and even transparency, among others. However, none of these products have reported using biopolymers in their manufacture.

## 6. Concluding Remarks

Nanofibers are a potential system to load natural bioactive compounds from herbal extracts to produce effective active matrices which can be used in healthcare therapies. Nanofibers’ properties such as high area surface, mesh porosity, and similarity to ECM architectural structure give this technology an exponential growth in its application in healthcare and biomedical engineering. The use of natural polymers enables the optimization of biocompatibility and biodegradability of these types of nanomaterials. This review has shown novel applications of active nanofibers in the pharmaceutical field such as antimicrobial, antioxidant, drug release, tissue engineering nanofibers, as well as in the food packaging industry. This review provided comprehensive information on the design and development of natural-based polymer nanofibers incorporated with the incorporation of herbal medicines for the treatment of common diseases and their in vivo studies. It is important to note the constant need to optimize technique parameters as well as material sources to improve and develop eco-friendly and effective alternatives for the healthcare area and food industry.

## Figures and Tables

**Table 1:** Influence parameters in nanofiber characteristics in Forcespinning^TM^.

Polymeric Solution Parameter	Technical Parameters	Environmental Parameters
Polymer viscoelasticity Polymer–solvent compatibility	Spinneret angular velocity Needle diameter Spinneret distance to the collector	Temperature Humidity

**Table 2:** Synthetic polymers used for nanofibers systems.

Polymer	Classification	Description	Reference
Poly (Glycolic Acid) (PGA)	Polyester	Thermoplastic polymer with high crystallinity (46–50%).	Park et al. [98]
		Transition and melting temperatures of 36 °C and 225 °C	
		Degradation product: glycolic acid	
Poly (Lactic Acid) (PLA)	Polyester	Semi crystalline polymer	Perumal et al. [99]; Zeng et al. [100]
		Hydrophobic	
		Degradation product: lactic acid	
Poly (Lactic-Glycolic Acid) (PLGA)	Polyester	Amorphous and crystalline polymer	Zhao et al. [101]
		Transition and melting temperature: 37 °C and 225 °C	
Poly (Ε-Caprolactone) (PCL)	Polylactone	Semi crystalline polymer	Ghosal et al. [102]
		Glass transition and melting temperature of −60 °C and 59 °C	
Polyvinyl Pyrrolidone (PVP)	Polyamide	Water-soluble polymer	Tsekova et al. [103]
		Glass transition temperature of 173 °C	
Poly (Vinyl Alcohol) (PVA)	Polyvinyl ester	Hydrophilic polymer	Chouhan et al. [104]
		Melting temperature of 300 °C	

**Table 3:** Natural herbal extracts incorporated into polymer nanofibers.

**Herbal Component**	**Polymer**	**Properties**	**Technique**	**Reference**
*Curcuma comosa* Roxb. Extract	Gelatin	Antioxidant, anti-tyrosinase, and anti-bacterial activities	Electrospinning	Chiu et al. [108]
*Lithospermi radix* extract	Gelatin/chitosan/PVA	Non-immunogenicity, antibacterial, tissue regeneration, anti-inflammatory, anti-apoptosis	Electrospinning	Yao et al. [109]
Pomegranate (*Punica granatum)* peel extract	Chitosan/polyethylene oxide (PEO)	Antioxidant, anti-diabetic, anti-hypersensitive, anti-inflammatory, antiviral, anti-bacterial	Electrospinning	Surendhiran et al. [105]
*Aloe vera* extract	Chitosan/polyethylene oxide (PEO)	Wound healing, anti-inflammatory, strengthening of the immune system, anti-carcinogenic, anti-diabetic, antioxidant	Electrospinning	Pathalamuthu et al. [110]
*Artemisia ciniformis* extract	PVA/chitosan	Antimicrobial	Electrospinning	Baniasadi et al. [111]
*Urtica dioica* L. extract	PCL	Antimicrobial	Electrospinning	Erbay et al. [112]
*Eleaeagnus angustifolia extract*	PEG-PCL-PEG	Antinociceptive, anti-inflammatory, antibacterial, antioxidant	Electrospinning	Hokmabad et al. [113]
Date palm fruit extract	PLA	Polyphenolic activity, antioxidant, anti-diabetic, anti-carcinogenic, antibacterial	Electrospinning	Zadeh et al. [114]
Copaiba (*Copaifera* sp.) oil	PLA/polyvinylpyrrolidone (PVP)	Anti-inflammatory, bactericidal	Solution blow spinning	Bonan, et al. [115]
*Lallemantia royleana* extract	PVA	Antioxidant, polyphenolic, and antimicrobial activities	Electrospinning	Rezaeinia et al. [116]
Grape Seed (*Vitis vinifera L.*) extract	PVA	Antioxidant	Electrospinning	Faki et al. [117]
*Juniperus chinensis*	PVA	Antibacterial, antifungal, antioxidant	Electrospinning	Kim et al. [118]
Lanasol from *Rhodomela confervoides*	PMMA/PEO	Antimicrobial	Electrospinning	Andersson et al. [119]
*Szygium aromaticum* extract	Thermoplastic polyurethane	Antibacterial, antiseptic, antifungal, analgesic, anticarcinogenic	Forcespinning	Canbay-Gokce et al. [120]
Tea tree oil extract (Melaleuca alternifolia)/Pomegranate peel extract	HP-ß-Cyclodextrin	Antioxidant, anti-inflammatory, antiseptic, and antimicrobial	Electrospinning	Kalouta et al. [121]
*Moringa oleifera* leaf extract	Polyacrylonitrile	Antimicrobial, antiproliferative, antioxidant, polyphenolic activity	Electrospinning	Fayemi et al. [122]
Resveratrol Veri-Te^TM^	Zein from maize	Antioxidant, anti-cancer, tissue engineering, barrier	Electrospinning	Leena et al. [123]
*Momordica charantia* fruit extract	Zein/gelatin	Antioxidant	Electrospinning	Torkamani, A. et al. [124]

## Data Availability

Not applicable.
